# Prognostic Impact of *Clostridium butyricum* MIYAIRI (CBM588) in Combination With Pembrolizumab for Advanced Urothelial Carcinoma: A Retrospective Cohort Study

**DOI:** 10.1002/cnr2.70308

**Published:** 2025-08-05

**Authors:** Junya Arima, Hirofumi Yoshino, Shuichi Tatarano, Akihiko Mitsuke, Yoichi Osako, Takashi Sakaguchi, Ryosuke Matsushita, Satoru Inoguchi, Yasutoshi Yamada, Hideki Enokida

**Affiliations:** ^1^ Department of Urology, Graduate School of Medical and Dental Sciences Kagoshima University Kagoshima Japan

**Keywords:** CBM588, pembrolizumab, probiotics, urothelial cancer

## Abstract

**Background:**

Combining the probiotic product CBM588 with immune checkpoint inhibitors (ICIs) has shown improved prognosis in several cancers. Pembrolizumab alone as a second‐line treatment for urothelial cancer (UC) extends prognosis by only 3 months.

**Aims:**

This is a retrospective study that examined the effects of CBM588 combined with pembrolizumab in advanced UC.

**Methods and Results:**

The study included 44 patients who had recurrence or progression after first‐line chemotherapy with cisplatin or carboplatin. The study compared 33 patients treated with pembrolizumab alone and 11 patients treated with CBM588 plus pembrolizumab over a median observation period of 12 months. The combination of CBM588 and pembrolizumab significantly improved progression‐free survival (PFS; *p* = 0.004) and overall survival (OS; *p* = 0.02). Multivariate analysis identified CBM588 as a significant prognostic factor for both PFS (hazard ratio: 0.074, *p* = 0.0008) and OS (hazard ratio: 0.105, *p* = 0.0056).

**Conclusion:**

These results suggest the effectiveness of the CBM588 combination with ICI treatment in UC.

## 1 Introduction

Cancer immunotherapy, including treatment with immune checkpoint inhibitors (ICIs), has had a major impact on treatment strategies for various cancers [1]. However, as with molecular‐targeted agents and anticancer therapy, immunotherapy approaches are limited by initial lack of efficacy of ICIs, low response rates to the therapy, and acquisition of resistance to ICIs in cancer cells [1]. Therefore, it is important to identify strategies that maximize the therapeutic effects of ICIs because these inhibitors can enhance antitumor effects by suppressing cells of the immune system.

The intestinal microbiota is known to have various functions, including food digestion, nutrient absorption, pathogen elimination, intestinal environment regulation, and vitamin production [2, 3]. The intestinal microbiota also plays an important role in host immunity [2, 4]. In addition, the gut microbiome has been shown to potentiate antitumor immune responses; therefore, it is possible that this may have a potential impact on the therapeutic efficacy of ICIs [5, 6, 7, 8]. For example, a clinical trial evaluating the efficacy of the probiotic product CBM588 in patients with metastatic renal cell carcinoma treated with the combination of nivolumab and ipilimumab showed that the addition of CBM588 extended PFS and OS [9]. These observations suggest that modulation of the gut microbiome may represent a promising approach to enhance the efficacy of cancer immunotherapeutic interventions.

UC is a cancer that arises from epithelial cells in the urinary tract, primarily in the renal pelvis, ureters, and bladder [10]. For years, platinum‐based chemotherapy was the mainstay for treating advanced and metastatic UC. In 2017, however, the US Food and Drug Administration approved pembrolizumab, an ICI, as a second‐line treatment or as a first‐line option for patients unfit for platinum‐based regimens [11, 12]. More recently, combined immunotherapy with enfortumab vedotin (EV; a microtubule inhibitor) and pembrolizumab was reported as a primary therapy for UC [13], highlighting the importance of ICIs in the treatment of UC. After the demonstration that CBM588 addition to ICI therapy prolongs survival in patients with renal cancer, several retrospective studies have reported similar results in gastric cancer and lung cancer [14, 15]. However, the usefulness of CBM588 combined with ICI treatment in UC is unknown.

Accordingly, the aim of our retrospective cohort study is to assess the prognostic significance of combining pembrolizumab with CBM588 in the treatment of UC.

## 2 Materials and Methods

### 2.1 Study Population

This retrospective cohort study was performed using clinical practice‐based databases from Kagoshima University Hospital in Japan. The study period was February 2018 to October 2024. Eligibility criteria included patients diagnosed with advanced UC—specifically, stage IV or recurrent metastatic disease—based on the Tumor‐Node‐Metastasis (TNM) classification system applicable to malignancies of the renal pelvis, ureter, and bladder [16]. This retrospective study analyzed 44 patients with advanced or recurrent metastatic UC who received second‐line pembrolizumab therapy—administered either as 200 mg every 3 weeks or 400 mg every 6 weeks intravenously—at Kagoshima University Hospital between January 10, 2018, and January 4, 2024. We divided the 44 patients into two groups: patients who received pembrolizumab alone and patients who received CBM588 when starting pembrolizumab treatment. CBM588 was prescribed in accordance with the drug information, at a dose of 3 g three times daily after each meal. Since we targeted patients who were already prescribed CBM588 at the start of pembrolizumab administration, the start date and indication of CBM588 varied among patients. This prescription was continued as long as pembrolizumab was administered. The treatment was continued until the following criteria were met: disease progression or withdrawal of consent by the patient. The treatment was also discontinued if unacceptable toxicity emerged. Reviewing medical records, we gathered the following data from the database: date; age; sex; Eastern Cooperative Oncology Group (ECOG) performance status (PS); initial diagnosis stage; liver, bone, or lung metastases; and appearance of immune‐related adverse events (irAEs). We excluded patients who were administered antibiotics orally or through infusion within 30 days before starting pembrolizumab or who were prescribed other probiotics. Patient characteristics are summarized in Table 1.

**TABLE 1:** Summary of patients characteristics.

		Pembrolizumab		Pembrolizumab + CBM588		*p*
		(*n* = 33)		(*n* = 11)		
Age, y.o.	Mean	72		72.3		0.914
	(Range)	(51–91)		(59–85)		
Race	Asian	33		11		
Sex	Male	23	69.70%	6	54.50%	0.468
	Female	10	30.30%	5	45.50%	
ECOG PS	0–1	13	39.40%	5	45.50%	0.738
	2–4	20	60.60%	6	54.50%	
Tumor location (primary tumor)	Upper urinary tract	19	57.60%	5	45.50%	0.509
	Lower urinary tract	14	42.40%	6	54.50%	
T stage	< 3	12	36.40%	4	36.40%	1
(Initial diagnosis)	≧ 3	19	57.60%	7	63.60%	
	Unknown	2	6.00%	0	0	
N stage	0	16	48.50%	4	36.40%	0.728
	≧ 1	17	51.50%	7	63.60%	
M stage	0	9	27.30%	4	36.40%	0.706
	≧ 1	24	72.70%	7	63.60%	
Liver metastasis	−	29	87.90%	9	81.80%	0.630
	+	4	12.10%	2	18.20%	
Bone metastasis	−	26	78.80%	9	81.80%	1
	+	7	21.20%	2	18.20%	
Lung metastasis	−	16	48.50%	8	72.70%	0.294
	+	17	51.50%	3	27.30%	
irAE	−	27	81.80%	9	81.80%	1
	+	6	18.20%	2	18.20%	

### 2.2 Outcomes and Variables

The primary outcomes were PFS and OS in the pembrolizumab‐only group compared with the pembrolizumab‐plus‐CBM588 group. CBM588 is usually prescribed for the treatment or prevention of diarrhea [17, 18], and this drug was taken three times daily. We also evaluated differences in clinicopathological factors between groups.

### 2.3 Statistical Analysis

Categorical and continuous variables were analyzed using Fisher's exact test and the Wilcoxon rank‐sum test, respectively. Covariate selection was informed by univariate Cox proportional hazards analysis, followed by multivariable Cox modeling. To assess multicollinearity, variance inflation factors (VIFs) were calculated; variables exhibiting high collinearity, such as lung and pancreatic metastases, were excluded from the multivariable analysis. Subsequently, Cox proportional hazards regression was utilized to estimate hazard ratios (HRs) and corresponding 95% confidence intervals (CIs) for PFS and OS. The Kaplan–Meier method was used to estimate the duration of pembrolizumab treatment, which ranged from 64 to 2071 days (median, 304 days). A two‐sided *p*‐value < 0.05 was considered statistically significant. All analyses were conducted using R (version 4.4.2; R Core Team).

## 3 Results

### 3.1 Patient Characteristics

**FIGURE 1:**
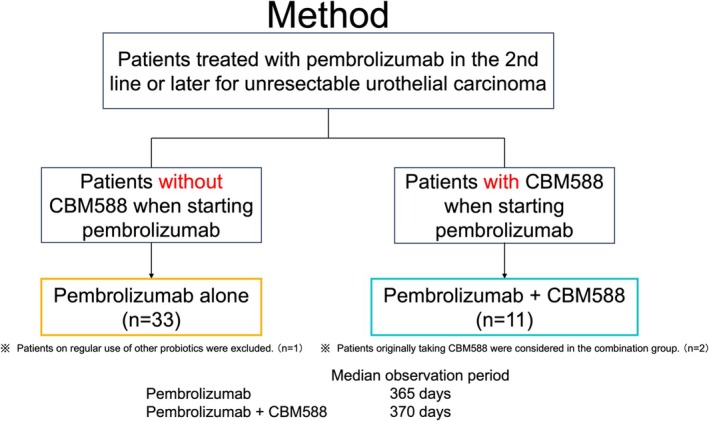
PRISMA (preferred reporting items for systematic reviews and meta‐analyses) showing the flow of participant enrollment and treatment.

We analyzed 44 patients' medical data retrospectively with advanced or recurrent UC consecutively treated with pembrolizumab alone or in combination with CBM588. Of these 44 patients, 25% (11 patients) were administered CBM588 when pembrolizumab was already started. Upper and lower urinary tract cancers accounted for 57.6% and 42.4% of cases, respectively, in the pembrolizumab alone group and 45.5% and 54.5% of cases, respectively, in the pembrolizumab plus CBM588 group. All patients in the combination group had already started receiving CBM588 at the initiation of pembrolizumab treatment. Characteristics of the patients are shown in Table 1. The median OS in our cohort was 12 months in both groups (Figure 1).

### 3.2 Efficacy Outcomes

**FIGURE 2:**
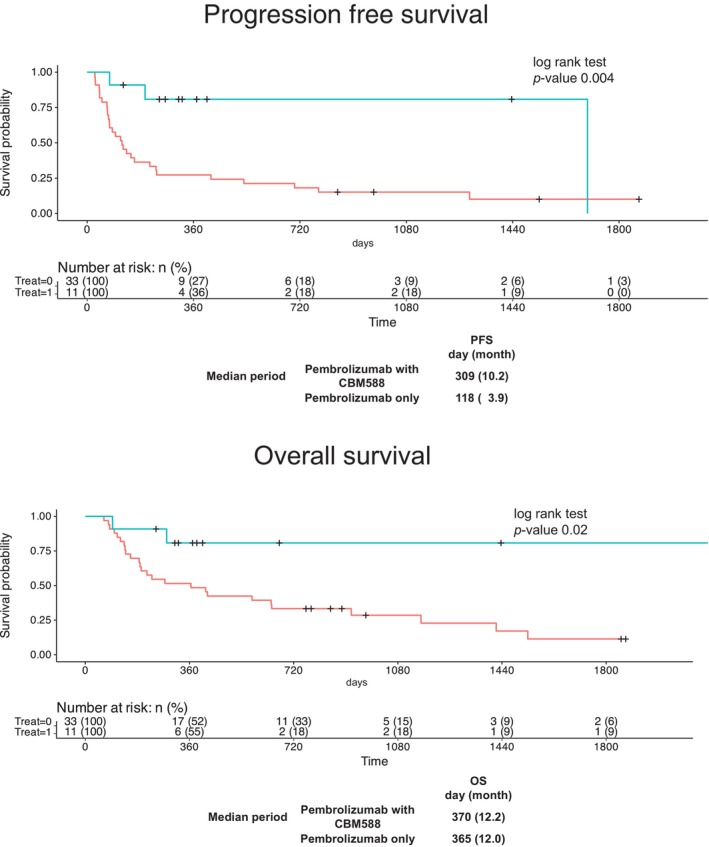
Efficacy outcomes in the treatment of patients with UC using pembrolizumab with or without CBM588. Progression‐free survival (a) and overall survival (b). Kaplan–Meier log‐rank tests were used to compare survival between the two arms.

Kaplan–Meier log‐rank tests showed that pembrolizumab plus CBM588 treatment significantly prolonged PFS compared with pembrolizumab alone (log‐rank test *p* = 0.004); the median PFS in the pembrolizumab alone group was 3.9 months, whereas that in the combination group was 10.2 months (Figure 2a). Kaplan–Meier log‐rank tests also showed that pembrolizumab plus CBM588 treatment significantly prolonged OS compared with pembrolizumab alone (log‐rank test *p* = 0.02); the median OS in the pembrolizumab alone group was 12.0 months, whereas that in the combination group was 12.2 months (Figure 2b). Multivariate analysis revealed that CBM588 was associated with longer PFS (HR, 0.074; 95% CI, 0.016–0.34; *p* = 0.0008) and OS (HR, 0.105; 95% CI, 0.022–0.533; *p* = 0.0056), as shown in Table 2. Furthermore, additional multivariate analyses revealed that more than T stage 3 (PFS: HR, 3.634; 95% CI, 1.340–9.835; *p* = 0.011) and (OS: HR, 3.9622; 95% CI, 1.210–8.763; *p* = 0.019) and liver metastasis (PFS: HR, 5.465; 95% CI, 1.516–19.70; *p* = 0.0094) were poor prognostic factors, whereas the appearance of irAE was a favorable prognostic factor (PFS: HR, 0.133; 95% CI, 0.03–0.586; *p* = 0.0077), consistent with previous reports.

**TABLE 2:** Multivariate analyses of progression‐free survival (PFS) and overall survival (OS) in patients with UC treated with pembrolizuma.

Variables	Groups	Multivariate analysis‐PFS				Multivariate analysis‐OS			
		Hazard ratio	95% CI	*p*		Hazard ratio	95% CI	*p*	
Treatment	PEM	Reference				Reference			
	PEM with CBM588	0.074	0.016–0.340	0.0008	**	0.105	0.022–0.533	0.0056	*
Age		0.947	0.901–0.995	0.03	*	0.992	0.944–1.043	0.738	
Sex	Male	Reference				Reference			
	Female	1.41	0.546–3.643	0.4786		0.717	0.292–1.985	0.513	
ECOG‐PS	0–1	Reference				Reference			
	2–4	0.338	0.136–0.837	0.0191	*	1.067	0.400–2.712	0.894	
Tumor location (primary tumor)	Lower urinary tract	Reference				Reference			
	Upper urinary tract	1.455	0.577–3.662	0.426		0.666	0.228–1.986	0.461	
T stage (initial diagnosis)	< 3	Reference				Reference			
	≧ 3	3.634	1.340–9.835	0.011	*	3.9622	1.210–8.763	0.019	*
	Unknown	7.623	1.291–45.00	0.025	*	6.368	1.069–37.93	0.042	*
N stage	0	Reference				Reference			
	≧ 1	0.976	0.380–2.505	0.958		1.328	0.521–3.389	0.552	
M stage	0	reference				reference			
	≧ 1	1.407	0.298–6.652	0.666		1.579	0.313–7.944	0.579	
Lung metastasis	+	0.871	0.297–2.55	0.801		0.497	0.151–1.633	0.249	
Liver metastasis	+	5.465	1.516–19.70	0.0094	*	2.889	0.735–11.34	0.129	
Bone metastasis	+	1.841	0.608–5.57	0.28		1.447	0.445–4.703	0.539	
irAE	+	0.133	0.03–0.586	0.0077	**	0.277	0.056–1.359	0.114	

*p < 0.05, **p < 0.01.

### 3.3 Safety

No patients in the CBM588 group experienced adverse reactions to CBM588, and no patients discontinued taking the drug. The irAE caused by Pembrolizumab was observed in 20% of patients. Appropriate measures such as Pembrolizumab withdrawal or treatment intervention were taken for patients who developed Grade 3 or higher irAE.

## 4 Discussion

The KEYNOTE‐045 and ‐052 trials evaluated the efficacy of pembrolizumab in patients with advanced or metastatic UC who had progressed to a metastatic condition after platinum‐based chemotherapy or primary treatment for platinum maladaptation, respectively [5, 12]. The results showed that pembrolizumab significantly improved OS compared with standard chemotherapy; however, this treatment only prolonged survival by 3 months. Recently, the KEYNOTE‐A39 (EV‐302) trial evaluated the combination of pembrolizumab and EV in patients with previously untreated locally advanced or metastatic UC and showed significant improvements in several key areas compared with standard chemotherapy, with a median OS of 31.5 months compared with 16.1 months for chemotherapy (HR, 0.47; 95% CI, 0.38–0.58; *p* < 0.0001), a median PFS of 12.5 months versus 6.3 months for chemotherapy (HR, 0.45; 95% CI, 0.38–0.54; *p* < 0.0001), and an objective response rate (ORR) of 68% with a complete response rate of 29%, compared to an ORR of 44% and a complete response rate of 12% for chemotherapy [13]. Therefore, ICI treatment is becoming increasingly important in the treatment of UC [19]. In this study, we showed that the combination of pembrolizumab with CBM588 as second‐line therapy may prolong survival, suggesting that the combination of pembrolizumab and EV with CBM588 may further improve the therapeutic effects of the combination. Further studies are needed to assess the effects of CBM588 in combination therapy with pembrolizumab and EV, and additional studies are needed to confirm the efficacy of CBM588 in combination with second‐line pembrolizumab therapy.

The combination of CBM588 with ICIs has been reported to improve treatment outcomes in various cancers [9, 14, 15]. In metastatic renal cell carcinoma, the addition of CBM588 to nivolumab‐ipilimumab combination therapy has been shown to extend survival [9]. The results also showed that there was an increase in regulatory T cells in the nivolumab and ipilimumab group but not in the group treated with CBM588. Furthermore, the levels of various cytokines, including C‐X‐C chemokine motif ligand 9, interleukin (IL)‐1β, granulocyte‐colony stimulating factor (CSF), IL‐10, IL‐12, granulocyte macrophage‐CSF, C‐C chemokine motif ligand 4, monocyte chemotactic protein 1, IL‐1RA, tumor necrosis factor‐α, IL‐2, IP‐10, IL‐2R, and IL‐8, were significantly increased in the CBM588 group [9]. A separate investigation revealed that CBM588‐derived butyrate, a short‐chain fatty acid, potentiates the antitumor activity of CD8^+^ T cells through an ID2‐dependent mechanism, achieved via suppression of histone deacetylase activity and subsequent upregulation of ID2 expression [20]. Conversely, exposure to antibiotics and PPIs has been demonstrated to induce alterations in the composition of the intestinal microbiota, which may consequently affect the therapeutic efficacy of ICI [21, 22, 23, 24]. Furthermore, a comparison of the fecal microbiota of patients who responded to ICI treatment showed that they had a greater variety of bacterial species than patients who did not respond [25]. These findings suggest that the gut microbiota has some influence on ICI treatment; however, the exact mechanisms through which the gut microbiota and probiotics alter the efficacy of ICIs remain unclear. Additionally, the efficacy of drugs altering the gut microbiota in combination with ICI treatment varies by region and study [26]; thus, it is unclear which specific microbiota are involved in the clinical efficacy of ICIs, and a consensus has not yet been reached. Notably, some reports have suggested that probiotic administration attenuates the clinical efficacy of ICI treatment [27]. Consequently, further research is necessary to provide more precise insights into the efficacy of these treatment strategies, thereby facilitating their clinical implementation.

In this study, we showed that the addition of CBM588 to pembrolizumab extended both PFS and OS. Furthermore, no adverse effects were observed with CBM588 administration, and no cases required treatment discontinuation. CBM588, which is already used as a probiotic in the clinical setting, has no specific precautions or contraindications and is relatively inexpensive, making it easy to prescribe. However, due to the imbalance in intestinal flora between men and women [28], it is imperative to acknowledge the influence of gender on ICI and gut microbiota intervention therapy. Although there is a certain bias in the urinary flora of bladder cancer patients, the characteristics differ depending on gender, and it is necessary to consider the differences in the effects of CBM588 [29]. Additionally, our cohort was limited to Japanese patients, was a small, single‐center study, and had a short observation period. Additionally, the analysis was retrospective. Given the limited sample size, the generalizability and robustness of our findings should be interpreted with caution. Therefore, larger, prospective, multicenter studies enrolling patients of different ethnicities and from different regions are required.

In conclusion, our findings demonstrated that the combination of CBM588 and pembrolizumab therapy extended both PFS and OS in patients with advanced or metastatic recurrent UC. Our findings suggest that CBM588 may influence the prognosis of UC patients receiving pembrolizumab therapy as a second‐line treatment. This approach suggests the applicability of other pembrolizumab‐based treatments. Further research is needed to verify these results and clarify the full extent of their therapeutic effects.

## Author Contributions

**Junya Arima:** conceptualization (equal), data curation (equal), formal analysis (equal), investigation (equal), methodology (equal), project administration (equal), resources (equal), software (equal), visualization (equal), writing – original draft (equal), writing – review and editing (equal). **Hirofumi Yoshino:** conceptualization (lead), data curation (lead), formal analysis (supporting), funding acquisition (lead), investigation (equal), methodology (equal), project administration (equal), resources (equal), software (equal), supervision (lead), validation (equal), visualization (equal), writing – original draft (equal), writing – review and editing (equal). **Shuichi Tatarano:** conceptualization (equal), formal analysis (equal), funding acquisition (equal), investigation (equal), methodology (equal), software (equal), supervision (equal), validation (equal), visualization (equal). **Akihiko Mitsuke:** data curation (equal), formal analysis (equal), investigation (equal), methodology (equal), resources (equal), software (equal). **Yoichi Osako:** conceptualization (equal), data curation (equal), formal analysis (equal), investigation (equal), resources (equal), software (equal), validation (equal). **Takashi Sakaguchi:** conceptualization (equal), data curation (equal), funding acquisition (equal), investigation (equal), methodology (equal), methodology (equal), resources (equal), resources (equal), software (equal), software (equal), supervision (equal), supervision (equal), validation (equal), validation (equal). **Ryosuke Matsushita:** formal analysis (equal), investigation (equal), methodology (equal), resources (equal), validation (equal). **Satoru Inoguchi:** data curation (equal), funding acquisition (equal), investigation (equal), methodology (equal), resources (equal), software (equal), validation (equal). **Yasutoshi Yamada:** data curation (equal), funding acquisition (equal), investigation (equal), methodology (equal), resources (equal), supervision (equal), validation (equal). **Hideki Enokida:** conceptualization (equal), funding acquisition (equal), methodology (equal), project administration (equal), supervision (equal), validation (equal), writing – original draft (supporting), writing – review and editing (supporting).

## Ethics Statement

This study was planned in accordance with the guidelines of the Declaration of Helsinki. This study was approved by the Bioethics Committee of Kagoshima University; written prior informed consent and approval were given by all patients prior to enrollment in the study. The approval number is 180308疫. The approved research period is from April 18, 2019 to March 31, 2024.

## Conflicts of Interest

The authors declare no conflicts of interest.

## Data Availability

The data that support the findings of this study are available from the corresponding author upon reasonable request.
